# Regulation of PBX3 expression by androgen and Let-7d in prostate cancer

**DOI:** 10.1186/1476-4598-10-50

**Published:** 2011-05-06

**Authors:** Håkon Ramberg, Ayham Alshbib, Viktor Berge, Aud Svindland, Kristin Austlid Taskén

**Affiliations:** 1 Faculty Division Aker University Hospital, University of Oslo, Oslo Urological University Clinic, Oslo University Hospital, Aker, N-0514 Oslo, Norway; 2 Faculty Division Aker University Hospital, University of Oslo, Department of Pathology, Oslo University Hospital, Aker, N-0514 Oslo, Norway; 3 Department of Tumor Biology, Institute for Cancer Research, Oslo University Hospital, Oslo, Norway

## Abstract

**Background:**

The pre-leukemia transcription factor 3 (PBX) is part of the PBX family of transcription factors, which is known to regulate genes involved in differentiation of urogenital organs and steroidogenesis. This is of interest with regard to prostate cancer progression as regulation of steroidogenesis is one of the mechanisms involved in the development of castration-resistant prostate cancer. In light of this we wanted to investigate the possible involvement of androgen regulation of PBX3 expression in prostate cancer.

**Results:**

In this study, we show that PBX3 is post-transcriptionally regulated by androgen in prostate cancer cells and that the effect might be independent of the androgen receptor. Furthermore, PBX3 was identified as a target of Let-7d, an androgen regulated microRNA. Let-7d was down-regulated in malignant compared to benign prostate tissue, whereas up-regulation of PBX3 expression was observed.

**Conclusions:**

We demonstrate that PBX3 is up-regulated in prostate cancer and post- transcriptionally regulated by androgen through Let-7d.

## Background

Transcription factors play a pivotal role in carcinogenesis due to their function as activators and repressors of gene expression. This key function also highlights their potential role as candidate drug targets and prognostic or diagnostic markers.

The Pre-B-cell leukemia transcription factors (PBX) are members of the TALE (three amino acid loop extension) homeobox gene family. They are involved in regulation of developmental gene expression, differentiation of urogenital organs and steroidogenesis through their abilities to form hetero-oligomeric DNA complexes [1,2]. PBX proteins interact with a subset of HOX proteins and with the Meinox subfamily of TALE class proteins to enhance their DNA-binding affinities and specificities. Human PBX1 was originally identified as a proto-oncogene in pre-B cell acute lymphoblastic leukemia where it is expressed as a fusion protein with E2A after a chromosomal translocation [3,4]. Later PBX2, PBX3 and PBX4 were identified as other members of the PBX family based on their high degree of sequence homology within and flanking their DNA-binding homeodomains [5,6]. Alternative splicing of PBX transcripts gives rise to high molecular weight (PBX1a, PBX2, PBX3a and PBX4) and low molecular weight (PBX1b and PBX3b, c, d) proteins [7].

Biochemical studies and expression profiling of PBX proteins indicate that they have both overlapping and specific functions [1]. Both PBX1 and PBX3 are expressed in the cortex of developing adrenal glands where they play a significant role in regulation of steroidogenesis [8,9]. More specifically, PBX has been shown to mediate ACTH-induced expression of CYP17A1 (cytochrome P-450 17alpha-hydroxylase), a key enzyme required for cortisol and androgen biosynthesis [10]. Members of the PBX family have also been shown to regulate metabolism of androgens in prostate cancer cells by modulating the expression of UGT2B17, an enzyme involved in glucuronidation of androgens [11].

PBX3 is highly expressed in developing central nervous system (CNS), but otherwise expressed at low level in the early phase of mouse organogenesis. Furthermore as a consequence of its role in the CNS, mice that are PBX3 deficient develop to term but die within a few hours due to central respiratory failure. Later, PBX3 becomes more widely expressed in epithelial and mesenchymal tissue throughout the embryo [12].

As regulation of steroidogenesis is one of the mechanisms involved in the development of castration-resistance prostate cancer, we wanted to study expression and regulation of PBX3 in prostate cancer. Furthermore, previous studies postulated that the PBX expression pattern could be used as a tool for both stratification and treatment of patients with cancer [13]. One possible treatment option is to use synthetic peptides that function as antagonists by blocking the HOX/PBX dimer formation. This approach has been reported to inhibit proliferation of ovarian, renal, non-small-cell lung cancer and pancreatic cancer cell lines [14-17].

## Results

### Androgen regulation of PBX3 at protein level in prostate cancer cell lines

**Figure 1:**
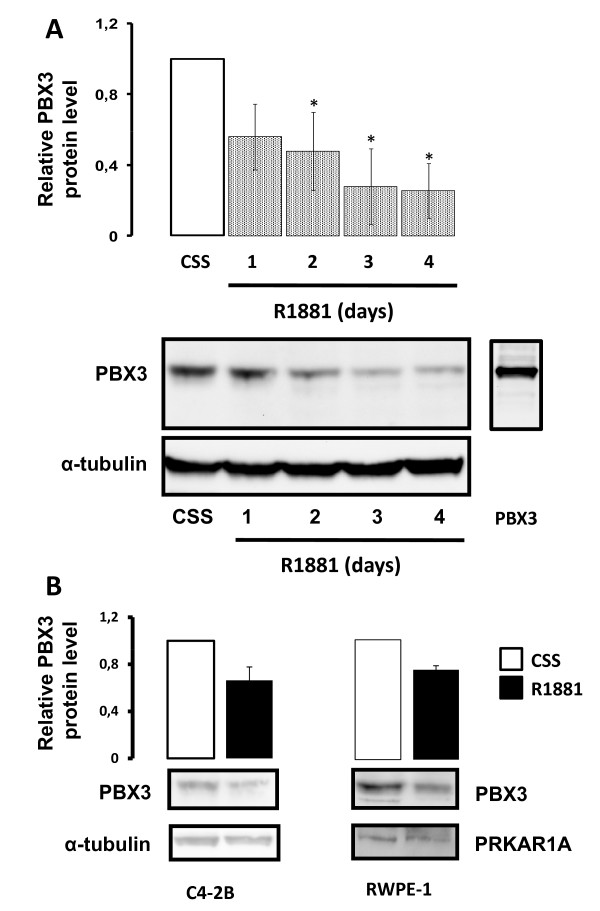
**Androgen regulation of PBX3 in prostate cancer cell lines**. LNCaP cells were stimulated with 10^-10^M R1881 (R1881) or left untreated (CSS) for 1 - 4 days before protein were extracted. **A**) Representative Western blots probed with anti-human PBX3 antibody (upper panel) and anti-α-tubulin antibody as loading control (lower panel) are shown. The lane to the right shows LNCaP cells transfected with a PBX3 expression plasmid (PBX3-SPORT6). Results of densitometric analysis of the PBX3 band relative to CSS are shown in the histogram above. **B**) Representative Western blots of C4-2B and RWPE-1 cells stimulated with 10^-10^M R1881 and probed with anti-PBX3 antibody are shown in the upper panels. Anti-α-tubulin antibody (C4-2B) or anti-PRKAR1A antibody (RWPE-1) are shown as loading controls (lower panels). Data were obtained from three independent experiments and are presented in the histograms as mean ± SD (n = 3). A t-test (paired two samples of mean) was performed and a two-tailed p-value < 0, 05 is indicated with a *.

In order to determine whether PBX3 expression is androgen regulated at the protein level, we stimulated LNCaP cells with 10^-10 ^M R1881, a synthetic androgen analogue, for 1 to 4 days followed by protein extraction and Western analysis using an anti-human PBX3 antibody. Down-regulation of PBX3 was detected after 24 hours of R1881 stimulation, and was further reduced after 4 days of treatment (Figure 1A). The androgen regulation of PBX3 was also observed in LNCaP C4-2B (C4-2B) and RWPE-1 cell lines (Figure 1B), and when LNCaP cells were stimulated with dihydrotestosterone (Additional file 1: Fig.S1). Over-expression of PBX3 by transfection of LNCaP cells (Figure 1A) and competition with a competitor peptide (data not shown) were used to confirm the specificity of the anti-PBX3 antibody. The level of PBX3 was unchanged in untreated LNCaP-cells during the experimental period (data not shown).

### Androgen receptor independent regulation of PBX3

**Figure 2:**
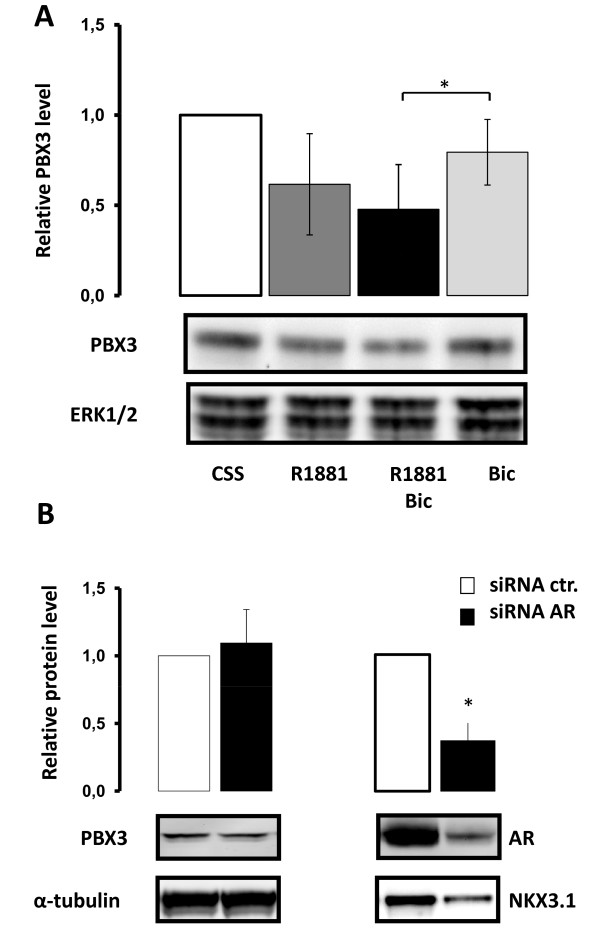
**Androgen receptor independent regulation of PBX3 by R1881**. **A**) LNCaP cells were pre-treated for 3 days with medium containing 10% CSS. Cells were then stimulated with 10^-10^M R1881 (R1881) and/or 10^-8^M bicalutamide (R1881 + Bic or Bic) or left untreated (CSS) for 4 days. Total protein extracts were analyzed by Western blotting using an anti-PBX3 antibody and anti-ERK 1/2 as loading control. Results of densitometric analysis of the PBX3 band relative to CSS are shown in the histogram. **B**) LNCaP cells were transfected with a small interfering RNA against androgen receptor (siRNA AR) and analyzed for PBX3 expression 48 hours post-transfection. A non-specific small interfering RNA (siRNA Ctr.) was used as control. Representative Western blots probed with anti-PBX3-, anti-AR-, anti-NKX3.1- and anti-α-tubulin antibodies are shown in the figure. The histograms depicts the results from densitometric analyses of PBX3 and AR relative to siRNA Ctr. Data were obtained from three independent experiments and are presented in the histograms as mean ± SD (n = 3). A t-test (paired two samples of mean) was performed and a two-tailed p-value < 0, 05 is indicated with a *.

In order to explore the role of the androgen receptor (AR) in androgen regulation of PBX3, LNCaP cells were stimulated with R1881 in the absence and presence of bicalutamide (Bic), an AR antagonist (Figure 2A). A minor reduction in PBX3 level was seen in the presence of bicalutamide alone and further reduction was observed by adding R1881. Transfection of LNCaP cells with siRNA targeting AR had no effect on the level of PBX3, whereas the level of AR itself and NKX3.1, a known androgen regulated gene, was reduced (Figure 2B).

### Post-transcriptional regulation of PBX3 by androgen

Interestingly, no significant change in PBX3 mRNA level was detected between control (CSS) and androgen-stimulated (R1881) LNCaP cells, and no effect of bicalutamide (Bic) was observed (Additional file 2: Fig.S2A). To determine whether androgen affects protein synthesis and/or degradation of PBX3 at the protein level, LNCaP cells were androgen stimulated in the presence or absence of cycloheximide, an inhibitor of protein synthesis. No significant effect of androgen was observed on PBX3 expression level when protein synthesis was inhibited. This indicates that androgen may affect translation/synthesis of PBX3 or that androgen indirectly regulates the protein stability of PBX3 (Additional file 2: Fig.S2B).

### Let-7d regulates PBX3 in LNCaP cells

**Figure 3:**
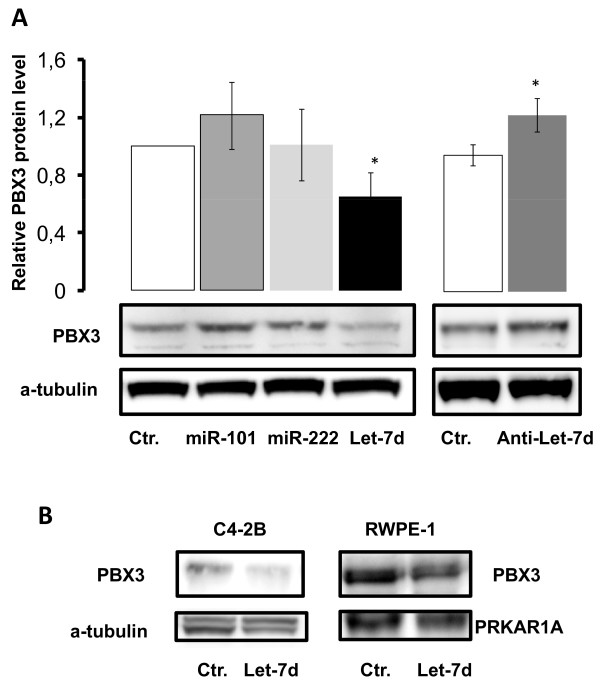
**MicroRNA regulation of endogenous PBX3 expression**. **A**) LNCaP cells were transfected with 5·10^-8^M miR-101, miR-222, Let-7d or control mimic microRNA (Ctr.) as shown in the left panel. The right panel shows transfection of LNCaP cells with 10^-7^M Let-7d mimic Hairpin Inhibitor. Total protein was extracted from the cells 48 hours post-transfection. Western analysis was performed with an anti-PBX3 antibody and anti-α-tubulin as loading control. Representative Western blots are shown and the results from the densitometric analysis of three independent experiments are presented in the histograms above (mean ± SD (n = 3)). A t-test (paired two samples of mean) was performed and a two-tailed P value < 0, 05 is indicated with a *. **B**) Let-7d mimic transfection of C4-2B and RWPE-1 cells followed by detection of endogenous PBX3 levels by Western analysis. Anti-α-tubulin (C4-2B) and anti-PRKAR1A (RWPE-1) antibodies were used as loading controls. Representative Western blots are presented.

One possible mechanism through which androgen may regulate protein synthesis of PBX3 is through microRNA that preferentially acts by binding to the 3' untranslated region (3'UTR) of specific mRNAs. MicroRNA that might regulate PBX3 expression was identified by *in silico *analyses using three different bioinformatic analysis software packages available, miRANDA, TargetScan and PicTar [18-20]. Putative binding sites for Let-7d, miR-101 and miR-222 were identified in the 3'UTR of PBX3 with all three bioinformatic programs (Table 1). Transfection of LNCaP cells with the three microRNAs identified *in silico *showed that Let-7d mimic decreased the endogenous protein level of PBX3 48 hours post-transfection, demonstrating that Let-7d potentially regulates PBX3 expression (Figure 3A). MiR-101 and miR-222 appeared not to have any effect on the level of PBX3 in LNCaP cells. A modest up-regulation of PBX3 was observed when transfecting LNCaP cells with a Let-7d mimic hairpin inhibitor (Anti-Let-7d), that blocks Let-7d microRNA (Figure 3A). Transfection of C4-2B and RWPE-1 cells with Let-7d mimic down-regulated the expression of endogenous PBX3 (Figure 3B).

**Table 1:** Predicted microRNA binding sites in PBX3 3'UTR region

microRNA	miRANDA	TargetScan	PicTar	Prostate Cancer
**miR-23a**	+	ND	ND	+
**miR-92 a/b**	+	ND	ND	+
**miR-101**	+	+	+	+
**miR-202**	+	ND	ND	+
**miR-222**	+	+	+	+
**miR-320**	ND	+	ND	+
**let-7a**	ND	+	ND	+
**let-7d**	+	+	+	+
**let-7g**	ND	+	ND	+

A list of differentially regulated microRNAs in benign and malignant prostate tissue was generated and potential binding sites in the PBX3 3'UTR region were identified using microRNA target prediction software. ND indicates that no binding site was detected/predicted by the software. The column marked Prostate Cancer indicates that the microRNA has been reported to be differentially regulated in benign and malignant prostate tissue.

**Figure 4:**
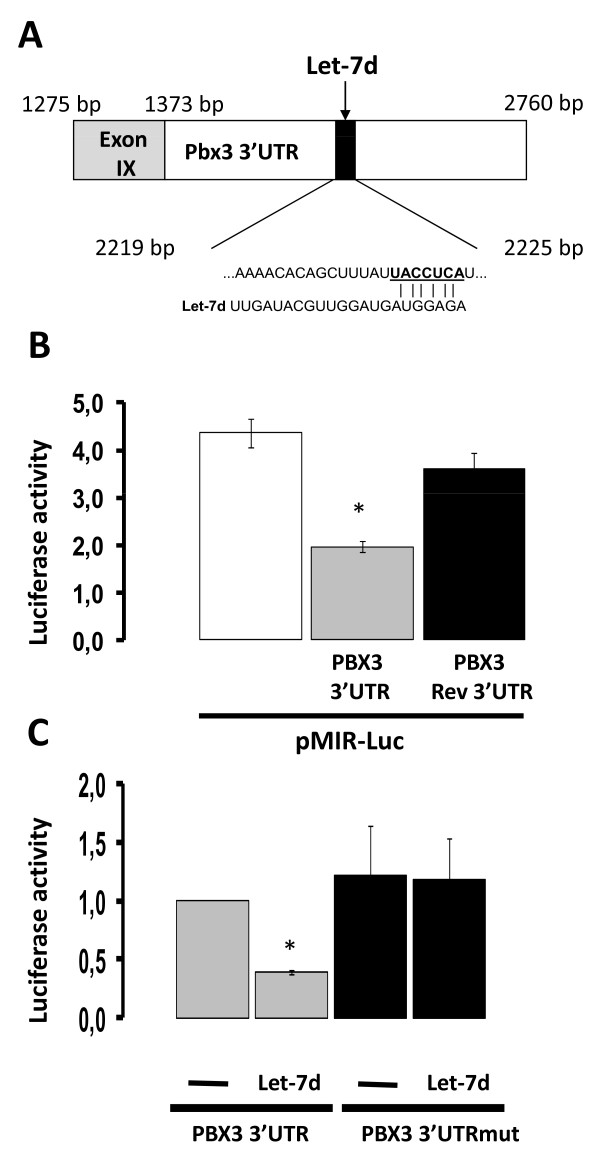
**PBX3 is a Let-7d target gene**. **A**) A schematic presentation of the 3'UTR sequence of PBX3 included in the reporter plasmids. The mature Let-7d sequence is shown underneath and the predicted Let-7d binding region is underlined. The underlined nucleotides are deleted in the pMIR-Luc-PBX3-3'UTR-Mut plasmid. **B**) LNCaP cells transiently transfected with either empty vector (pMIR-Luc) or reporter plasmids containing the 3'UTR-PBX3 in either the correct (pMIR-Luc-PBX3 3'UTR) or the inverse orientation (pMIR-Luc-PBX3 3'UTR-Rev) were harvested and reporter activity measured. **C**) Reporter plasmids containing the 3'UTR of PBX3 with either the wild-type binding site of Let-7d present (pMIR-Luc-Pbx3 3'UTR marked PBX3 3'UTR) or deleted (PBX3 3'UTRmut) were transfected into LNCaP cells in the absence or presence of 5·10^-8 ^M Let-7d mimic. The cells were harvested 48 hours post-transfection. Luciferase activity adjusted for β-galactosidase activity is shown in the histograms. Data are presented as mean ± SD (n = 3) relative to LNCaP cells transfected with PBX3 3'UTR. A t-test (paired two samples of mean) was performed and a two-tailed p-value < 0, 05 is indicated with a *.

The putative Let-7d binding site in the 3'UTR region of PBX3 is depicted in Figure 4A. The 3'UTR sequence of PBX3 was cloned into the pMIR-Luc plasmid and transfected into LNCaP cells. The presence of the PBX3 3'UTR (pMIR-Luc-PBX3-3'UTR) reduced the luciferase activity compared to an empty reporter plasmid (pMIR-Luc), as would be expected if microRNA regulated the expression of PBX3 (Figure 4B). No significant effect was observed when the 3'UTR PBX3 sequence was cloned into pMIR-Luc in the reverse orientation (pMIR-Luc-PBX3-Rev3'UTR). To test whether Let-7d regulated PBX3 expression, the reporter construct, pMIR-Luc-PBX3-3'UTR, was co-transfected with Let-7d mimic into LNCaP cells. Co-transfection of Let-7d mimic reduced the luciferase activity generated by pMIR-Luc-PBX3-3'UTR (Figure 4C). The effect of Let-7d was blocked by mutating the putative binding site of Let-7d in the 3'UTR sequence of PBX3 (PBX3 3'UTRmut).

### Androgen regulation of Let-7d in prostate cancer cell lines

**Figure 5:**
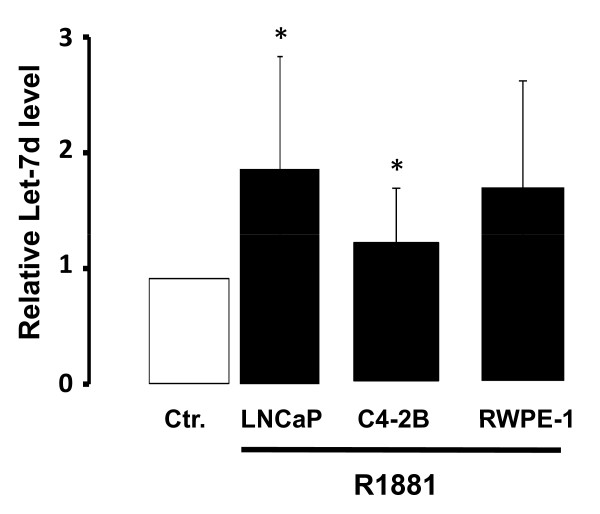
**Androgen regulation of Let-7d in prostate cancer cell lines**. LNCaP, C4-2 and RWPE-1 cells were stimulated with 10^-10^M R1881 (R1881) or left untreated (Ctr) for 24 hours. Let-7d levels were quantitated using sqRT-PCR. RNU6B microRNA was used for normalization. Data are presented as mean ± SD (n = 3) relative to the corresponding untreated cells. A t-test (paired two samples of mean) was performed and a two-tailed P value < 0, 05 is indicated with a *.

Androgen regulation of PBX3 via Let-7d was further explored by measuring Let-7d levels after R1881 stimulation. LNCaP, C4-2B and RWPE-1 cells were stimulated with 10^-10 ^M R1881 for 24 hours and the level of Let-7d was measured by sqRT-PCR. As shown in Figure 5 there was an up-regulation of Let-7d after 24 hours of androgen treatment relative to unstimulated cells.

### PBX3 expression and Let-7d levels in prostate specimens

**Figure 6:**
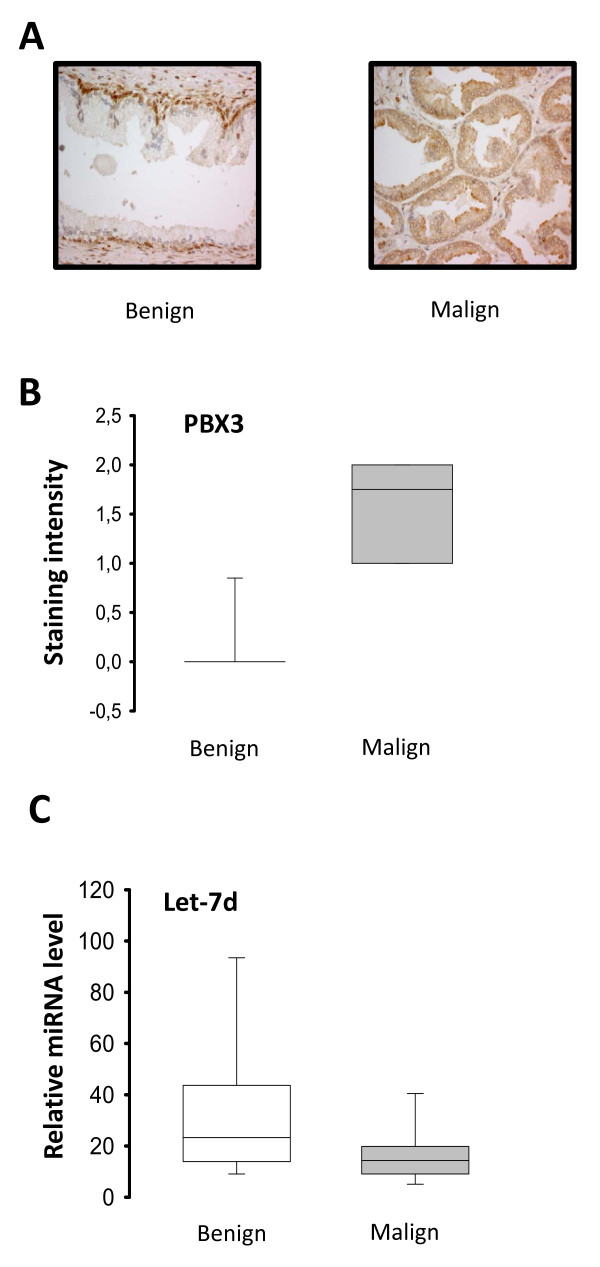
**Expression of Let-7d and PBX3 in human prostate cancer specimens**. **A**) Representative images of benign and malignant prostate tissue immunohistochemically stained with anti-human PBX3 antibody. **B**) TMA slides, with benign (n = 21) and malignant (n = 30) prostate tissue cores, were immunohistochemically stained with anti PBX3 antibody and scored according to staining intensity from 0 to 3. **B**) Levels of Let-7d in benign and malign prostate tissue samples (n = 18) from Oslo Urological University Clinic were quantified by sqRT-PCR. RNU6B microRNA was used for normalization. A Pearson's chi-squared test was performed showing a significant difference (p < 0.001). Vertical lines in each box represent the median value and Whiskers show the 95% confidence interval.

Tissue micro-array slides with 21 benign and 30 malignant tissue cores from patient's having undergone laparoscopic prostatectomy at Oslo Urological University Clinic (Norway) were analyzed for PBX3 expression by immunohistochemical staining as shown in Figure 6A. PBX3 was detected in the nucleus of basal cells in benign areas, whereas cytoplasmic expression of PBX3 was observed in malignant prostate cancer. Scoring of the staining intensities showed that the level of PBX3 was up-regulated in malignant compared to benign tissue (Figure 6B). A Pearson's chi-squared test was preformed showing a significant difference (p < 0.001) between the PBX3 expressions in benign compared to malignant prostate cancer tissue.

We observed an up-regulation of PBX3 at the  protein level in malignant prostate cancer, whereas analysis of Let-7d  expression in 18 matched benign and malignant samples from prostatectomy specimens showed down-regulation of Let-7d in malignant compared to benign tissue samples (Figure 6C).

## Discussion

This study shows that PBX3 is post-transcriptionally regulated by androgen in prostate cancer cell lines. Furthermore, PBX3 is identified as a target of Let-7d, an androgen regulated microRNA, indicating that androgen regulates PBX3 expression through Let-7d. PBX3 was up-regulated and Let-7d was down-regulated in prostate cancer compared to normal epithelial prostate cells.

Androgen regulation of PBX3 was predominantly post-transcriptional since no regulation of PBX3 at the mRNA level following androgen stimulation was detected. This is in contrast to the regulation of PBX1, previously identified as a target gene for PLZF (promyelocytic leukemia zinc finger), an early androgen responsive gene [21]. Kikugawa and colleagues showed that PBX1 was regulated after androgen treatment in DU145 cells transfected with the androgen receptor, indicating that PBX1 was indirectly regulated by androgens through PLZF. Whereas putative PLZF-binding sites are present in the PBX1 promoter [22], a preliminary analysis using the TFSEARCH software by our group generated no putative PLZF-binding sites in the upstream region of the human PBX3 gene (data not shown) [23]. Previous studies have shown that all-trans retinoic acid induced the expression of PBX1 at mRNA level in P19 embryonic carcinoma cells, whereas the PBX3 mRNA level remained unchanged. The protein level of PBX3 was, however, induced by all-trans retinoic acid [24,25].

Interestingly, our data indicate that androgen regulates the expression of PBX3 independently of AR as the R1881 effect was observed in LNCaP cells transfected with siRNA targeting AR and in RWPE-1 cells that express very low levels of AR (not detectable on Western blot) [26]. The androgen effect was also seen in the presence of bicalutamide, an anti-androgen. Although most effects of androgens are mediated by AR, some responses like rise in intracellular calcium and cAMP concentrations seem to take place independent of AR in some cell types [27]. Further studies are needed to explore the molecular mechanisms involved in AR independent effects of androgens.

Since androgen had no effect on the PBX3 mRNA level or protein stability in our study, we hypothesized that androgen might regulate the protein synthesis of PBX3 through microRNA. MicroRNAs are short oligonucleotide, 28-24 nucleotides long, that binds to the 3' untranslated region (3'UTR) of their target genes. The binding of a mature microRNA to the RNA-inducing silencing complex (RICS) can initiate translational inhibition and RNA degradation. Putative binding sites for three microRNAs, miR-101, miR-222 and Let-7d were identified in the 3'UTR region of the PBX3 gene by using three different microRNA target prediction software. In this study, we have focused on Let-7d, since transfection with Let-7d mimic, in contrast to transfection of miR-101 and miR-222 mimics, reduced the protein level of endogenous PBX3 in LNCaP cells.

Let-7d is a microRNA that is part of the Let-7 family [28]. Many of the 13 Let-7 family members have been shown to be regulated in prostate cancer [29-33]. Interestingly, Let-7d was recently identified as an androgen-regulated microRNA in LNCaP and LAPC-4 cell lines following microarray analysis, giving support to our data indicating androgen regulation of PBX3 synthesis through Let-7d [34]. The Let-7 family is involved in normal development and differentiation, and has been shown to act as tumor suppressors in many human cancers [28]. In LNCaP cells, Let-7a was recently shown to have an anti-proliferative effect [35]. We were able to detect reduced viability in LNCaP cells transfected with Let-7d (data not shown), demonstrating the possible tumor suppressor function of the Let-7 family in prostate cancer, a function that is supported by the observed down-regulation of Let-7d expression in malignant compared to benign prostate cancer tissue. Two studies have previously reported down-regulation of Let-7d in malignant compared to benign prostate tissue using microarray analysis [29,30] whereas a third study by Volinia et.al. showed an up-regulation of Let-7d in malignant prostate tissue [36].

Whereas Let-7d expression was reduced in prostate cancer, increased expression of PBX3 was observed in malignant versus benign prostate tissue by immunohistochemical staining of prostate specimens with an anti human PBX3 specific monoclonal antibody. We observed nuclear staining of basal cells in benign prostate tissue and no detectable staining of luminal cells. However in malignant tissue, staining was detected both in the cytoplasm and nucleus of secretory luminal cells. Previous work by Crijns and colleagues [37] reported increased PBX immunohistochemical staining in both cytoplasm and nuclei of ovarian tumor cells using an anti-PBX1/2/3/4 antibody, which does not differentiate between the different PBX members. Future work will show whether PBX3 has a potential role as a biomarker or drug target in prostate cancer as shown in other types of cancer [14-17].

## Conclusions

Here we demonstrate for the first time that PBX3 is up-regulated in malignant prostate tissue. The presented results show that androgen regulates PBX3 expression via Let-7d in the prostate cancer cell lines tested. This is of interest in light of the role of androgens in prostate cancer progression and the role of microRNA in regulating gene expression at the post-transcriptional level. The putative involvement of PBX in regulation of differentiation and steroidogenesis makes the PBX members interesting candidates for therapeutic targeting in later stages of prostate cancer. It would therefore be of interest to establish whether disruption of the PBX transcription complex formation using PBX-binding peptides like HXR9, can halt progression of advanced stages of prostate cancer.

## Materials and methods

### Cell culture

LNCaP and RWPE-1 cells were purchased from ATCC (Rockville, MD) and cultured in RPMI 1640 medium supplemented with 10% (v/v) fetal bovine serum (FCS) or Keratinocyte-SFM medium from Invitrogen (Carlsbad, CA, USA) supplemented with 2.5 μg Epidermal Growth Factor (EGF) and 25 mg Bovine Pituitary Extract (BPE), respectively. The LNCaP C4-2B cell line was kindly provided by professor Leland W.K. Chung at Emory University, Atlanta, Georgia. C4-2B cells were cultured in T-medium (D-MEM:F-12 (1:1) + L-Glutamin+15 mM HEPES (Invitrogen, Carlsbad, CA) supplemented with 5 μg/ml insulin, 0,0136 ng/ml T3, 5 μg/ml apo-transferin, 0,25 μg/ml biotin, 25 μg/ml adenine, 50 μg/ml streptomycin, 50 μg/ml penicillin and 5% FCS). All the cell lines were maintained at 37°C in a 5% CO_2_-humidified incubator. CSS was prepared by charcoal treatment of FCS under agitation at 4°C overnight, followed by centrifugation and sterile filtration. R1881/Methyltrienolone was purchased from Roussel-UCLAF (Romainville, France) and bicalutamide was purchased from Astra Zeneca (Cheshire, UK). All other compounds were from Sigma (St. Louis, MO) unless otherwise specified.

### Stimulation of prostate cancer cell lines

LNCaP and C4-2 were pre-treated for 3 days (Figure 1, 2) or 24 hours (Figure 5) with medium containing 10% CSS before stimulation. The cells were treated with 10^-10^M R1881 (synthetic androgen), 10^-8^M bicalutamid and/or 10 μg/ml cycloheximide. Cycloheximide and bicalutamide was added 2 hours before R1881. Cells incubated in 10% CSS throughout the experiment were used as controls. RWPE-1 cells were not pre-treated since they are cultivated in serum free medium.

### Semi-Quantitative Real-Time RT-PCR (sqRT-PCR)

Total RNA was isolated using Trizol™ from Invitrogen (Carlsbad, CA,). 100 ng of total RNA were included in a one-step RT-PCR reaction using QuantiTect SYBR Green RT-PCR kit from Qiagen (Hilden, Germany) that was performed using MJ Research DNA Engine Opticon Continuous Fluorescence Detection System from MJ Research Inc. (Walthan, MA). RT-PCR cycle conditions were as follows; reverse transcription at 50°C for 30 min and inactivation step 95°C for 15 min, followed by 40 PCR cycles (15 s at 94°C, 30 s at 55°C, 30 s at 72°C), and a final extension at 72°C for 5 min followed by a melting curve analysis. G6PD was used for normalization. The ΔΔCt formula was used as described in the protocol from Applied Biosystems (Foster City, CA). The following primers were used; *G6PD *[NM_000402]: tgcatgagccagataggc and acagggaggagatgtggttg; *PBX3 *[NM_006195]: gcattaatcattacatcg and tgacagttcagggcatgttt. The Taqman microRNA assay from Applied Biosystems was used to detect and quantitate Let-7d expression. 10 ng of total RNA were used in a two step RT-PCR reaction with the hsa-Let-7d Taqman^® ^MicroRNA Assay kit (Applied Biosystems, Cat. no 4395394), Taqman^® ^MicroRNA Reverse Transcription kit (Applied Biosystems, Cat. no 4366596) and Taqman^® ^2X Universal PCR Master Mix (Applied Biosystems, Cat. no 4324018) in accordance with the manufacturer's protocols. The RNU6B Taqman^® ^MicroRNA Assay (Applied Biosystems, Cat. no 4373381) was used for normalization. The ΔΔCt and ΔCt formulas were used as described in the protocol from Applied Biosystems (Foster City, CA).

### Protein extraction and Western analysis

Cells were harvested in PBS and lysed in RIPA buffer (25 mM Tris-HCl pH 7.6, 150 mM NaCl, 1% NP-40, 0.1% SDS) and Complete protease inhibitor Tablets from Roche (Mannheim, Germany) followed by 30 min rotation at 4°C. The lysate was centrifuged at 10,000 g for 30 min and the amount of protein in the supernatant was estimated by Bradford protein assay kit from Bio-Rad Laboratories (Hercules, CA). Protein extracts (50 μg/lane) and MagicMark (Invitrogen Carlsbad, CA) were separated on NuPAGE 10% or 4-12% gradient Bis-Tris gels from Invitrogen (Carlsbad, CA) and transferred to PVDF membranes purchased from Millipore Corporation (Billerica, MA) by wet electro blotting. Membranes were blocked in 5% (w/v) skimmed milk powder in Tris-buffered saline containing 0.1% (v/v) Tween-20 (TBST) for 2 hours at room temperature (RT), and then incubated over night at 4°C with the appropriate primary antibody diluted in TBST. Primary antibodies used were mouse monoclonal anti-PBX3 antibody from Abnova Corporation (Heidelberg, Germany), anti-PRKAR1A antibody from Transduction Laboratories (Franklin Lakes, NJ), anti-ERK 1/2 from Cell Signaling Technology (Boston, MA) and mouse monoclonal anti-α-Tubulin antibody from Sigma (St. Louis, MO). The NKX3.1 antibody was kindly provided by professor Fahri Saatcioglu at Department of Molecular Biosciences, University of Oslo, Norway. Membranes were washed 3 × 10 min with TBST and further incubated for 1 hour with anti mouse or anti rabbit IgG horse-radish peroxidase-conjugated secondary antibodies from Jackson ImmunoResearch Ltd (Cambridgeshire, UK). After washing 3 × 10 min, membranes were developed using Immobilon™ Western by Millipore (Billerica, MA). Images were generated using a Hamamatsu Digital Camera from Syngene (Cambridge, UK), and quantified using the Multi Gauge software from FujiFilm (Bedford, UK).

### Prostate tissue specimen

Matched benign and malignant prostate tissues for mRNA analysis and immunohistochemical staining were derived from radical prostatectomy of 18 and 30 prostate cancer patients, respectively, treated at Oslo Urological University Clinic and obtained from "The Prostate Biobank - a resource for urological research in Norway" (10974). The tissue samples were treated with RNALater at 4°C overnight according to the manufactures protocol (Qiagen, Hilden, Germany) and stored at -80°C. The pathology of the samples was verified by an experienced uropathologist (AS). Written consent was obtained from all patients and the project was approved by The Regional Committee for Medical and Health Research Ethics.

### Immunohistochemistry

Immunohistochemical staining of paraffin embedded tissue sections were performed according to the manufactures protocol using Ultravision One detection system from LabVision (Fremont, CA) and IHC Select manual staining system from Chemicon (Temecula, CA). Tissue microarray (TMA) slides were deparaffinised and antigen retrieved in citrate buffer (pH 6) for 30 min at 100°C in the PT module from Lab-Vision (Suffolk, UK). The TMA slides were incubated with anti human PBX3 antibody (dilution 1:50). Non-immune IgG from Vector Laboratories Inc. (Burlingame, CA) was used as negative control. Slides were treated for 70 s in haematoxylin. Manual scoring of the staining was done by an experienced pathologist who also verified all pathological gradings (AS). The tissue microarray of specimens from radical prostatectomies contained benign and malignant tissue from 30 patients. Images were captured using Leica DM RA2 microscope and Leica DC 300F camera.

### Plasmids

The PBX3-SPORT6 expression vector containing a full length cDNA clone of PBX3 from Imagene (Imagene, IRATp970H04107D) was used for overexpression of PBX3. The pMIR-REPORT™ miRNA Expression Reporter Vector System (Applied Biosystems, Cat. no AM5795) consisting of pMIR-REPORTER™ Luciferase and pMIR-REPORTER™ β-gal Control Plasmids was used in this study. The former (pMIR-Luc) was used to generate luciferase reporter constructs with the PBX3 3'untranslated regions (nucleotides 1275-2760 NM_006195). The following primer set, PBX3 3'UTR; agacgggagctcatcaatcagacgggaggctac and agaataaagcttcattagaatcaccgccacaa, was used to generate a PBX3-3'UTR amplicon from the PBX3-Sport6 vector and cloned into the pMIR-Luc repoter vector. A reporter vector with the PBX3 3'UTR sequence in the reverse orientations was also designed with the PBX3 Rev3'UTR primer set; tgggactagtcctcattagaatcaccgccacaa and tcttataagctttcaatcagacgggaggctac. The GeneAmp High Fidelity PCR system from Applies Biosystems was used according to the manufacturer's protocol. The predicted Let-7d seed binding region, tacctca, of PBX3 3'UTR was deleted using the QuickChange ll Site Directed Mutagenesis kit (Stratagene, cat. No 200523). The protocol from the manufacturer was followed and the two mutagenic deletion primers designed and used in the construction of the pMIR-PBX3-3'UTR-Mut were ccaatcaaaacacagctttattgcgaactcatacaaacc and ggtttgtatgagttcgcaataaagctgtgttttgattgg. All the plasmids constructed were verified by sequencing at GATC Biotech (GATC Biotech, Germany).

### Transfection and Luciferase assay

Cells were seeded on either Petri dishes (10 cm) at 1 × 10^6 ^cells per dish, 6 wells plates at 300 000 cells per well or 96 well-plates at 5000 cells per well and cultured for 3 days in complete medium. Cells were transfected with the plasmids mentioned above. Small interfering RNA (siRNA) against AR (Dharmacon, Cat no. KROAA-005852) and miRNA hsa-Let-7d miRIDIAN Mimic (Dharmacon, Cat. no C-300478-07), hsa-Let-7d miRIDIAN Mimic Hairpin Inhibitor (Dharmacon, Cat. no C-300478-08) and miRIDIAN microRNA Negative control 1 (Dharmacon, Cat. no CN-001000-01-05). DharmaFECT™ Duo Transfection reagent (Dharmacon Cat. no. T- 2010-03), DharmaFECT™ 3 (Dharmacon Cat. no. T- 2003-03) or Lipofectamine 2000 (Invitrogen) were used according to the manufactures protocols with minor modifications. Cells were incubated in medium with serum and without antibiotics for 2 hours before transfection. The transfection medium was changed after 4 hours of transfection to medium with serum and without antibiotics and further incubated for 24 or 48 hours. Luciferase activity was measured using a Luciferase assay system (cat. no.1501, Promega, Madison, WI). The beta-galactosidase assay of the pMIR-REPORTER™ β-gal Control Plasmid was performed with Galacto Plus and Emerald reagents (Applied Biosystems). We followed the protocol according to the manufacturer. We used a Luminometer (TD-20/20 Turner Designs, Sunnyvale, CA) to measure the luciferase and β-galactosidase activities.

## Competing interests

The authors declare that they have no competing interests.

## Authors' contributions

HR took part in designing the study, performed experiments and analyzed results, as well as writing the draft and final manuscript. AA studied androgen regulation of PBX at mRNA and protein level in untransfected prostate cancer cell lines, and took part in drafting of the manuscript. VB was involved in the study design and statistical analysis of immunohistochemistry results and revision of the final draft. AS evaluated the morphology of the tissue samples, scored the immunohistochemical staining and participated in the revision of the final draft. KAT designed the study, analyzed the experimental results and took part in writing of the draft and revision of the final manuscript. All authors have read and approved the final draft of the manuscript.

## Supplementary Material

Additional file 1 **Figure S1. Regulation of PBX3 protein level by DHT in LNCaP**. **A**) LNCaP cells were pre-treated for 3 days with 10% CSS and stimulated with dihydrotestosterone (DHT) with different concentrations as indicated in the figure for 48 hours. The Western blot was probed with an anti-PBX3 antibody. **B**) A representative Western blot of LNCaP cells stimulated with 10^-10 ^M R1881 for 1-4 days using an anti-PBX1/2/3/4 antibody. Click here for file

Additional file 2 **Figure S2. PBX3 is post-transcriptionally regulated by androgen**. LNCaP cells were pre-treated for 3 days with medium containing 10% CSS. Cells were then stimulated or left untreated (CSS). **A**) Total RNA was extracted after 48 hours of 10^-10^M R1881 (R1881) and/or 10^-8^M bicalutamide (R1881 + Bic or Bic) treatment to determine the effect of R1881 on the expression levels of PBX3 mRNA by sqRT-PCR relative to CSS. **B**) LNCaP cells were either left untreated (CSS), stimulated with 10^-10^M R1881 (R1881) alone or in combination with 10 μg/ml cycloheximide (R1881 + Cyc) for 48 hours. Cells only stimulated with cycloheximide (Cyc) are shown as control. The cells were pre-treated with cycloheximide for 2 hours before adding 10^-10^M R1881. Western blots were probed with anti- PBX3 antibody and densitometrically analysed. Anti α-tubulin antibody was used as loading control. Data is presented as mean ± SD (n = 3). Click here for file
