# Supplementary figures and images for: Regulation of PBX3 expression by androgen and Let-7d in prostate cancer
Source: Mol Cancer. 2011 May 6;10:50. doi: 10.1186/1476-4598-10-50 (PMC3112428; doi:10.1186/1476-4598-10-50)

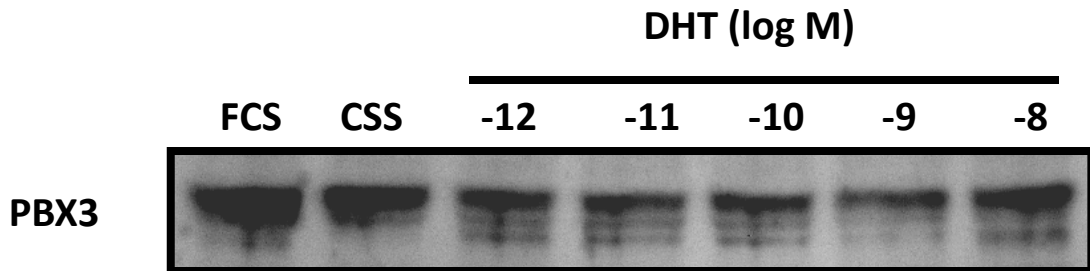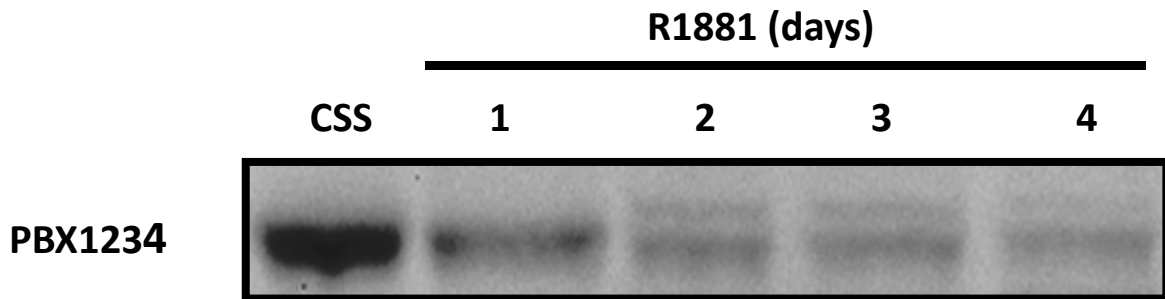

Supplement: Additional file 1 — Figure S1. Regulation of PBX3 protein level by DHT in LNCaP. A) LNCaP cells were pre-treated for 3 days with 10% CSS and stimulated with dihydrotestosterone (DHT) with different concentrations as indicated in the figure for 48 hours. The Western blot was probed with an anti-PBX3 antibody. B) A representative Western blot of LNCaP cells stimulated with 10-10 M R1881 for 1-4 days using an anti-PBX1/2/3/4 antibody. [file 1476-4598-10-50-S1.PDF]

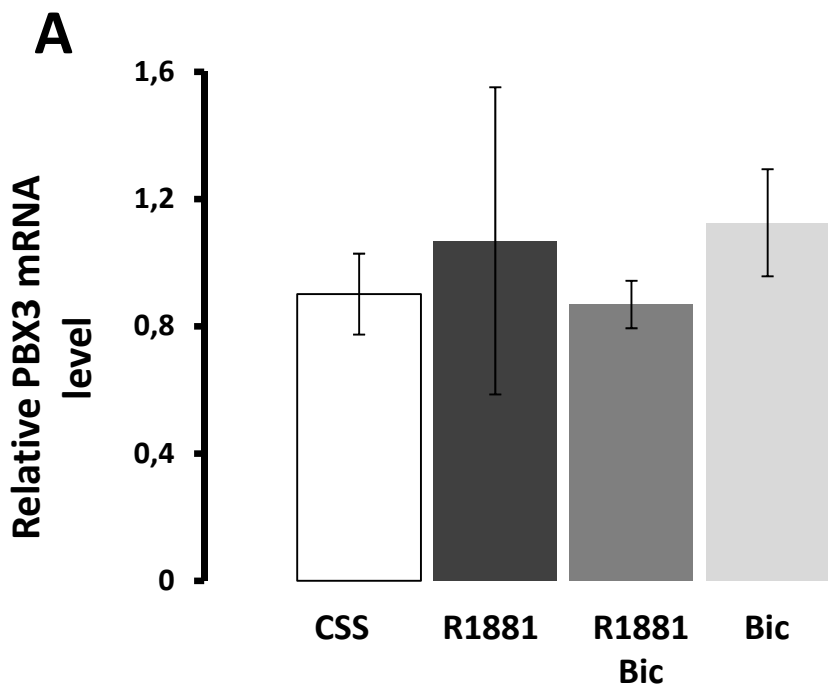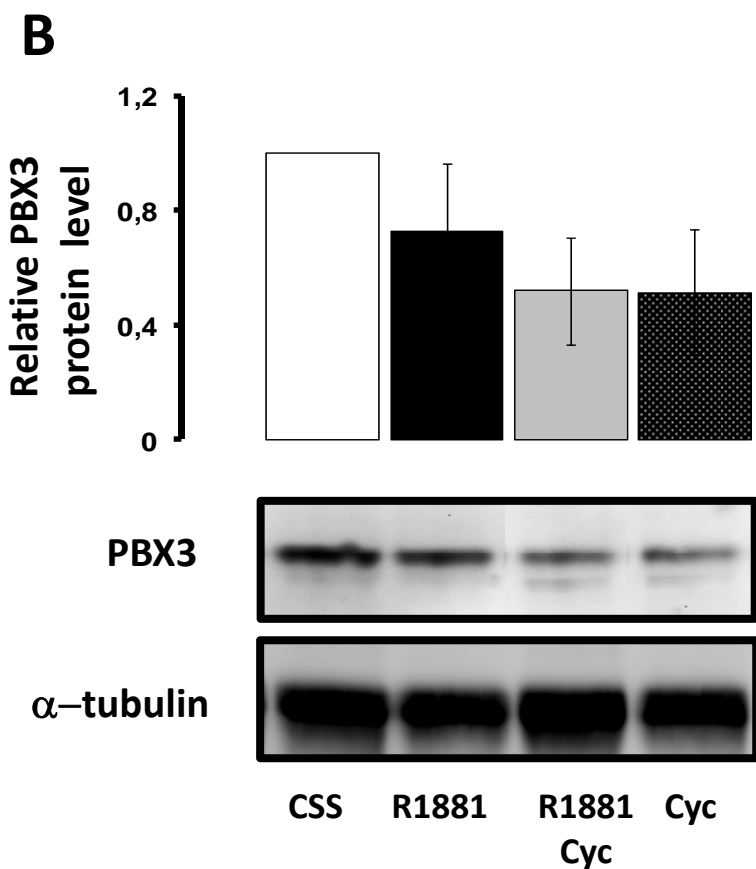

Supplement: Additional file 2 — Figure S2. PBX3 is post-transcriptionally regulated by androgen. LNCaP cells were pre-treated for 3 days with medium containing 10% CSS. Cells were then stimulated or left untreated (CSS). A) Total RNA was extracted after 48 hours of 10-10M R1881 (R1881) and/or 10-8M bicalutamide (R1881 + Bic or Bic) treatment to determine the effect of R1881 on the expression levels of PBX3 mRNA by sqRT-PCR relative to CSS. B) LNCaP cells were either left untreated (CSS), stimulated with 10-10M R1881 (R1881) alone or in combination with 10 μg/ml cycloheximide (R1881 + Cyc) for 48 hours. Cells only stimulated with cycloheximide (Cyc) are shown as control. The cells were pre-treated with cycloheximide for 2 hours before adding 10-10M R1881. Western blots were probed with anti- PBX3 antibody and densitometrically analysed. Anti α-tubulin antibody was used as loading control. Data is presented as mean ± SD (n = 3). [file 1476-4598-10-50-S2.PDF]
